# Malaria Screener: a smartphone application for automated malaria screening

**DOI:** 10.1186/s12879-020-05453-1

**Published:** 2020-11-11

**Authors:** Hang Yu, Feng Yang, Sivaramakrishnan Rajaraman, Ilker Ersoy, Golnaz Moallem, Mahdieh Poostchi, Kannappan Palaniappan, Sameer Antani, Richard J. Maude, Stefan Jaeger

**Affiliations:** 1 Lister Hill National Center for Biomedical Communications, National Library of Medicine, National Institutes of Health, Bethesda, MD 20894 USA; 2 Institute for Data Science and Informatics, University of Missouri-Columbia, Columbia, MO 65211 USA; 3 Electrical and Computer Engineering Department, Texas Tech University, Lubbock, TX 79409 USA; 4 Electrical Engineering and Computer Science Department, University of Missouri-Columbia, Columbia, MO 65211 USA; 5 Mahidol Oxford Tropical Medicine Research Unit, Mahidol University, Bangkok, 10400 Thailand; 6 Centre for Tropical Medicine and Global Health, Nuffield Department of Medicine, University of Oxford, Oxford, UK; 7 Harvard TH Chan School of Public Health, Harvard University, Boston, USA

**Keywords:** Automated light microscopy, Smartphone application, Malaria, Machine learning, Convolutional neural network

## Abstract

**Background:**

Light microscopy is often used for malaria diagnosis in the field. However, it is time-consuming and quality of the results depends heavily on the skill of microscopists. Automating malaria light microscopy is a promising solution, but it still remains a challenge and an active area of research. Current tools are often expensive and involve sophisticated hardware components, which makes it hard to deploy them in resource-limited areas.

**Results:**

We designed an Android mobile application called Malaria Screener, which makes smartphones an affordable yet effective solution for automated malaria light microscopy. The mobile app utilizes high-resolution cameras and computing power of modern smartphones to screen both thin and thick blood smear images for *P. falciparum* parasites. Malaria Screener combines image acquisition, smear image analysis, and result visualization in its slide screening process, and is equipped with a database to provide easy access to the acquired data.

**Conclusion:**

Malaria Screener makes the screening process faster, more consistent, and less dependent on human expertise. The app is modular, allowing other research groups to integrate their methods and models for image processing and machine learning, while acquiring and analyzing their data.

## Background

Microscopic examination of stained blood smears is still considered the gold standard for malaria diagnosis [1, 2]. It offers the ability to characterize parasite species, quantify parasite density, and assess the effectiveness of antimalarial treatment. However, regions that are suffering from the disease are often lacking in well-trained personnel that can perform high-quality microscopy examination due to the high costs to train such experts [3, 4]. Besides, the examination process can be very time-consuming and error-prone.

To address these issues, there have been attempts to automate both image acquisition and image analysis for the microscopic examination of blood smears. Gopakumar, G.P. et al. [5] proposed a custom-built portable slide scanner that automatically collects and analyzes focus stacks of blood smear images. Muthumbi, A. et al. [6] proposed a system that adds a programmable LED array to the standard microscope, and uses a large-field-of-view, low-resolution objective lens to capture thousands of cells in one snapshot. While these methods show great potential, they are often hard to test on a large scale, especially in resource-limited settings, due to the difficulty to replicate their sophisticated hardware design. Other research work [7–9] concentrates on image analysis algorithms. They tend to be lacking a user interface to put their systems to use in real clinical settings.

**Fig. 1:**
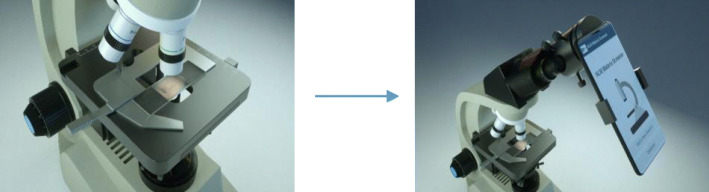
System Setup for Malaria Screener. During the (semi-) automated* screening process, the body of the smartphone is attached to an adapter. The adapter holds the phone, and aligns its camera with the eyepiece of the microscope. * The system is semi-automated in that the user needs to move the slide manually to search for an ideal field of view while capturing smear images

In this paper, we present a smartphone-based semi-automated system that provides analysis of blood smear images for malaria screening, with an easy-to-use user interface. Our Android smartphone application combines multiple functions, including image acquisition, image screening, and management of the acquired data. The smartphone is used in combination with a microscope adapter as shown in (Fig. 1), which is a very affordable setup by design. Android smartphones and microscopes are commonly available in malaria clinics, and an adapter is usually inexpensive. For example, the universal smartphone microscope adapter we use costs less than $100 (from *telescopeadapters.com*, model: USPA2). The low-cost design and easy-to-use interface give the system great potential to assist malaria diagnosis in resource-limited areas. Furthermore, the modular architecture allows it to be adapted by fellow researchers to advance their study.

## Implementation

### Software architecture

**Fig. 2:**
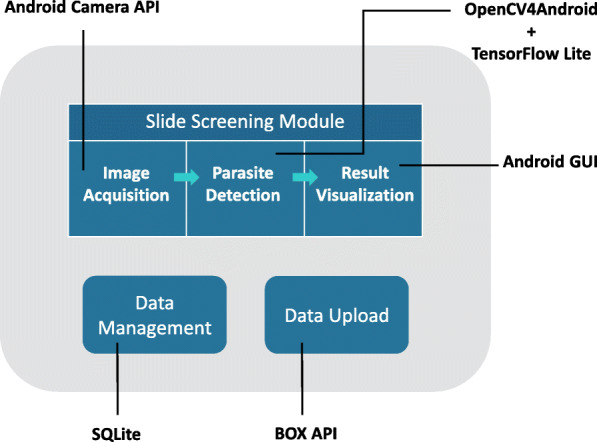
Diagram of the application software architecture and interfaces

We designed Malaria Screener by following object-oriented principles. A diagram of the application’s architecture is shown in Fig. 2. It consists of three independent modules, including: a slide screening module, a data management module, and a data upload module. The slide screening module, being the core of the system, has three sub-modules that work sequentially to perform image acquisition, parasite detection, and result visualization, respectively. The data management module stores the images and the corresponding metadata acquired during slide screening sessions, giving user access to previously screened slides. Finally, the data upload module transfers the local data to an online repository for record-keeping and further training of the system. We implemented the front end user interface (UI) based on Android API while the back-end of the application is powered by a mix of different libraries including OpenCV4Android (opencv.org/android/), TensorFlow Lite [16], SQLite [18], and Box API [19].

Malaria Screener is designed to be easily extendable and customizable. The source code is hosted on GitHub as an open-source project; fellow researchers can modify the code to suit their needs. For example, developers can replace the parasite detection module to test their detection algorithm, or can add another classifier to detect other malaria parasite species

### Critical components

#### Slide screening module

As mentioned above, three independent sub-modules work sequentially to screen a slide for malaria parasites. The image acquisition module is the first module in this pipeline. For this module, we implemented a customized camera function using the Android Camera API [10]. This includes a Camera object that controls the intrinsic parameters of the camera hardware, and a CameraPreview object that displays the preview image to the user. During a screening session, the user presses a button to capture the image when a suitable field of view becomes visible. To obtain the best image quality possible, the Camera object requests the maximum resolution that the smartphone camera offers and saves the captured image as PNG, a lossless compression format.

**Fig. 3:**
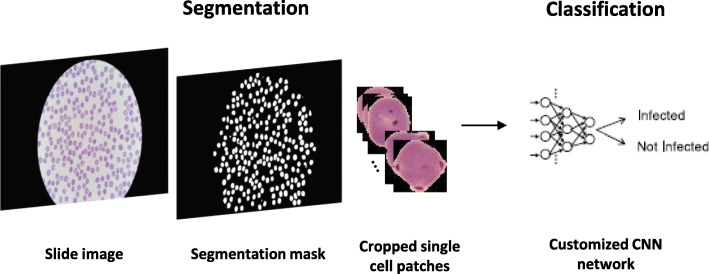
Diagram of the parasite detection module for a thin smear input. The original image is first segmented using a watershed algorithm to extract single-cell patches. These cell patches are then classified by a customized CNN model, which has been pre-trained using TensorFlow framework, and deployed on the smartphone with TensorFlow Lite

The captured image is then passed to the parasite detection module as input. Malaria Screener can examine both thin and thick smears with potential *P. falciparum* infections. The performance evaluation of the detection module can be found in previous publications [11–14]. Figure 3 illustrates how an image is processed in this module. For a single thin smear image, the goal is to detect the number of infected red blood cells (RBCs) and the total number of RBCs in the image. For a thick smear image, on the other hand, the goal is to detect the number of parasites and white blood cells (WBCs). The parasite detection module has a *ThinSmearProcessor* class and a *ThickSmearProcessor* class to handle each of the two scenarios. With *ThinSmearProcessor, a* thin smear image is first segmented to detect RBCs. Small cell patches of RBCs are cropped from the original image. With *ThickSmearProcessor*, parasite candidate patches that cover the typical size of a parasite are cropped from a thick smear image. Both classes use pre-trained Convolutional Neural Network (CNN) models to make binary classifications: infected vs uninfected RBC in the case of a thin smear, or parasite vs background in the case of a thick smear. The CNN models are pre-trained on a PC with TensorFlow and Keras, which outputs the trained models in HDF5 (.h5) format. Next, the models are converted to Protocol Buffers (.pb) format [15] and deployed to the app using TensorFlow Lite [16, 17].

The result visualization module uses *ResultDisplayerActivity,* a UI class that was implemented to present the detection results to the user (Fig. 5 (a)(3)). This class generates a down-sampled version of the captured image with labels drawn on the infected RBCs (parasites for thick smear images). In addition, it uses a table to show the numerical results. Together, these two outputs visualize the computational result of the input smear for the user.

#### Data management module

**Fig. 4:**
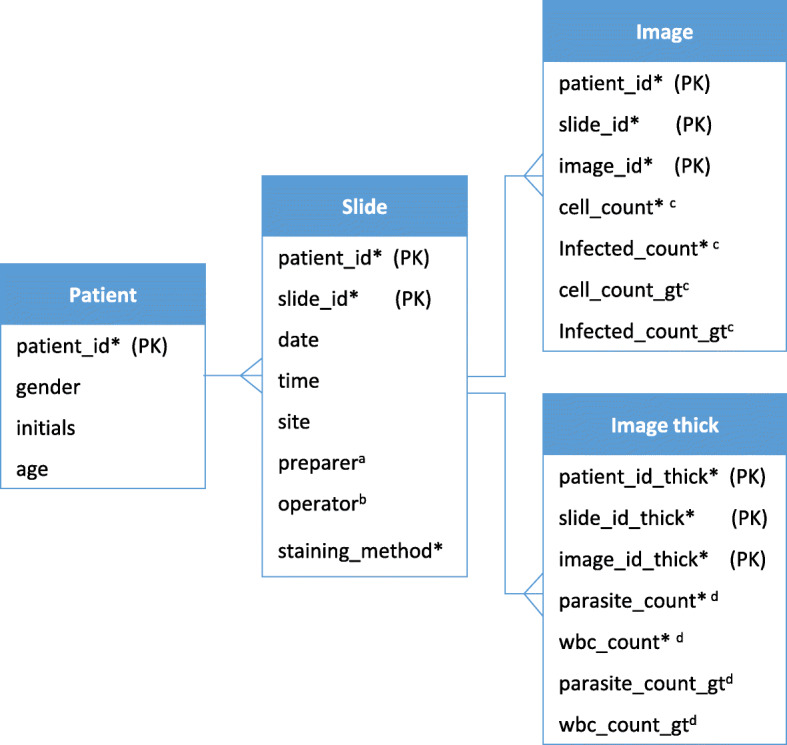
Diagram of the local SQLite database. PK: primary key. Each line that connects two tables indicates the one-to-many relationship between them. For example, the patient table has a one-to-many relationship with the Slide table, meaning one patient can have multiple slides. Fields with an asterisk symbol (*) are either mandatory inputs by the user or automatically generated data; other fields are optional inputs by the user. ^*a*^ *Name of the slide preparer.* ^*b*^ *Name of the user performing the screening.* ^*c*^ *App outputs and manual counts for thin smears: RBC counts, infected RBC counts, manual RBC counts, manual infected RBC counts.* ^*d*^ *App outputs and manual counts for thick smears: parasite counts, WBC counts, manual parasite counts, manual WBC counts*

**Fig. 5:**
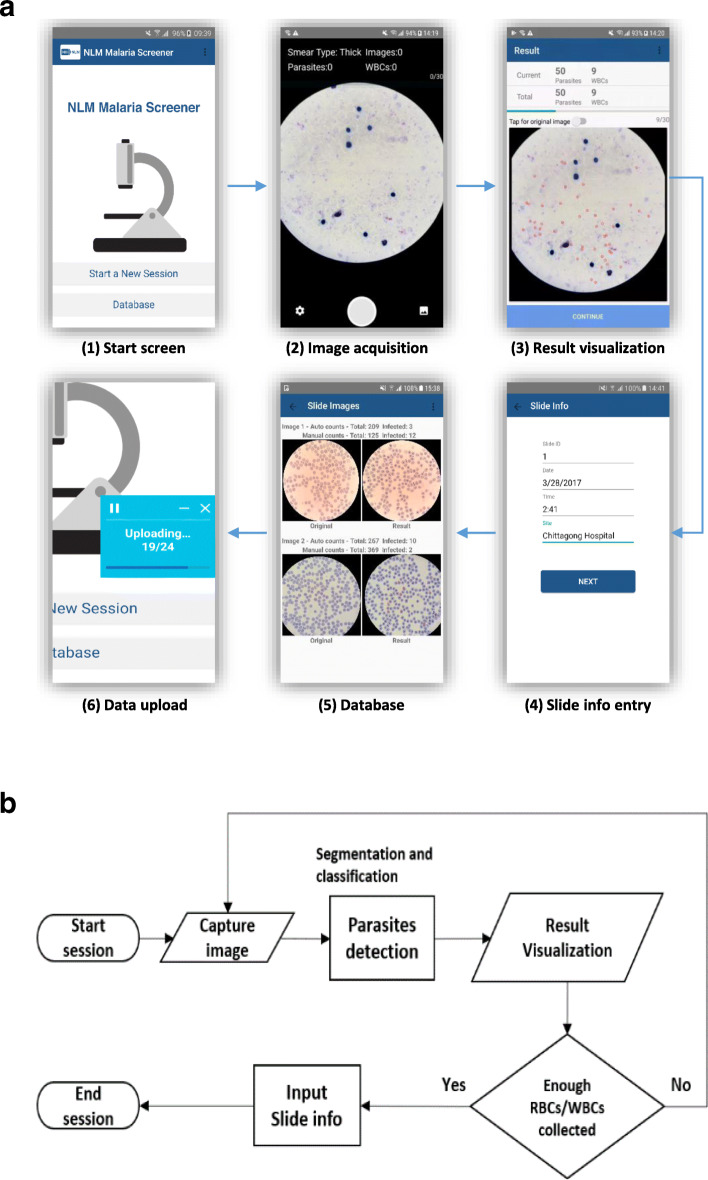
**a** UI screens during a slide screening session. **b** The workflow of a session

The app stores images and corresponding metadata locally on the phone. Images are stored in a designated folder of the internal storage. Within this folder, images from the same screening session are grouped in their own sub-folders. Metadata of the images is stored in a local SQLite database [18]. The database includes four tables: a patient table, a slide table, a thin smear image table, and a thick smear image table. Figure 4 shows the structure of the database in more detail. The data management module also offers a UI to let the user browse the images and metadata stored in the SQLite database, as shown in (Fig. 5 (a)(5)).

#### Data upload module

Images and metadata in the database can be exported and uploaded to an online repository. The uploaded data can be used to examine the app performance, and to improve the classifier of the parasite detection module with additional training.

An upload event can be initiated in two different ways. The first option is to start an upload event from the database UI. With this option, the app will scan for all data that has not been uploaded yet, which will then be uploaded. However, this type of bulk upload can be a very heavy task, which can take a long time since there might be several gigabytes of images to be uploaded. Therefore, we implemented another upload option in which the app attempts to trigger an upload event after each screening session. As long as a Wi-Fi connection can be detected, this event will try to upload the data from the current session.

The back-end of this module is implemented with a mixture of both Android and Box API [19]. Android Service class and Thread class [20, 21] are used to implement the upload function which executes the upload tasks in a separate thread. It allows the user to continue with other things while the upload tasks proceed in the background. Box API is used to implement functions to execute upload tasks to a Box repository.

## Results

A fast and effective mobile app is developed as a lightweight solution to automated malaria light microscopy. This section describes its workflow during a slide screening session. Due to space constraints, this section only illustrates the important parts of the screening pipeline. For more details, readers should refer to the user manual, which can be downloaded together with the source code, see download link in *Availability of Data and Materials*. The workflow proceeds in six steps as follows, with each step corresponding to a panel in (Fig. 5 (a)):

1. Once the smartphone is setup on top of the microscope, using an adapter, the user can start a session from the main page of the app.
2. A preview screen of the camera is presented to the user at the beginning of a session, and the user can use the button at the top of the screen to set the smear type (thin or thick) at this point. Then, the user can search for a suitable field of view on the slide, and press the camera button to capture the image.
3. The app will then start to process the captured image, and will display a result visualization page on the screen where it shows the detection results. For example, in thin smear mode, the number of infected and total RBCs are shown as well as a running total. The app also shows a result image with the infected RBCs marked in red. Step (2) and (3) are repeated (Fig. 5 (b)) while the user captures more smear images. The iteration stops when the total number of RBCs reaches a user-determined maximum.
4. Next, the app goes through several screens to let the user enter relevant information about the slide, such as slide ID, staining method, and hematocrit value.
5. Then, the session ends. Both the images and metadata are saved locally, and can be viewed through the database UI at a later time.
6. Finally, the app triggers an upload event to send the saved data to the central Box repository. Meanwhile, a floating widget hovers over the app screen to show the upload progress.

### Testing

Tests were performed with the algorithms we implemented for the slide screening module. We acquired and annotated Giemsa-stained thick and thin blood smear images from 150 patients infected with *P. falciparum,* and from 50 normal patients, at Chittagong Medical College Hospital, Bangladesh.

For thick smear, we evaluated the performance of our system with five-fold cross validation, using 2967 thick blood smear images from these 200 patients: 1819 images from 150 infected patients and 1148 images from 50 normal patients. Table 1 shows the mean performance of our system on five folds at both patch and patient level [12].

**Table 1:** System mean performance on five folds for thick smears

	Accuracy	AUC	Sensitivity	Specificity	Precision
Patch-level [12]	96.89%	98.48%	90.82%	97.43%	74.84%
Patient-level [12]	78.00%	84.90%	79.33%	74.00%	90.42%

For thin smear, we also performed five-fold cross validation at both patch and patient level [14]. We compared our results with the state-of-the-art on patch level, as shown in Table 2. To the best of our knowledge, we could find no comparable literature that performed cross-validation studies on patient-level.

**Table 2:** Classification module mean performance on five folds for thin smears compared to the state-of-the-art

	Accuracy	AUC	Sensitivity	Specificity	F1-score
Proposed Module (Patch-level) [14]	**98.6%**	**99.9%**	**98.1%**	**99.2%**	**98.7%**
Proposed Module (Patient-level) [14]	95.9%	99.1%	94.7%	97.2%	95.9%
Gopakumar et al. (2018) [5]	97.7%	–	97.1%	98.5%	–
Bibin, Nair & Punitha (2017) [22]	96.3%	–	97.6%	95.9%	–
Dong et al. (2017) [23]	98.1%	–	–	–	–
Liang et al. (2017) [24]	97.3%	–	96.9%	97.7%	–
Das et al. (2013) [25]	84.0%	–	**98.1%**	68.9%	–
Ross et al. (2006) [26]	73.0%	–	85.0%	–	–

More details can be found in our previous publications [11–14, 27]. We are currently in the process of field testing our app with collaborators around the world.

## Discussion

Malaria Screener is a step towards automating malaria light microscopy. It provides a solution to improve malaria point-of-care diagnosis in the field. To the best of our knowledge, Malaria Screener is the first smartphone-based system that can screen thin and thick smears. In addition to the basic slide screening functions, which are based on computational image analysis and machine learning, we try to integrate additional functions into our mobile app to support the daily work of malaria field workers. In particular, the data management function can be very helpful. Users can enter patient information directly into the app’s database, thus avoiding the trouble of using a separate system to manage the data.

For malaria research, the app offers a powerful and efficient tool for field tests and data collection, which are usually done through a collaboration between medical imaging research groups and hospitals. Coordinating the protocols typically requires a considerable effort, involving data processing and formatting. Malaria Screener solves this problem by integrating a slide screening module, a database module, and a data upload module into the same smartphone application, making slide screening and data collection a streamlined process that generates and delivers ready-to-use data.

Finally, with the release of the current codebase of the software as an open-source project, we anticipate it to serve groups that are new to this field of research. The modular design allows other developers to build upon the current implementation. For example, our parasite detection algorithm can be easily swapped, allowing other groups to test their own algorithms. By making Malaria Screener an open-source project, we are hoping to provide a platform for the scientific community to work together and to advance the automation of malaria diagnosis.

## Conclusions

We present a fast, low-cost smartphone application for malaria screening. We demonstrate that the app offers important functionalities with an intuitive user interface to (a) screen slides and count infected red blood cells and parasites in thin and thick smear images automatically for *P. falciparum* malaria, and (b) to manage the images and metadata generated throughout the screening process, which can be used to further optimize the image analysis model.

Based on the promising results from previous tests, and interest shown by the research community, we anticipate this project to serve as a code base for future developments in this area.

### Availability and requirements

Project name: Malaria Screener.

Project home page: https://lhncbc.nlm.nih.gov/project/malaria-screener

Operating System: Android.

Programming language: Java, C++ (for Android Native development).

Other requirements: Android Lollipop/5.0 and above.

License: Open Source Software.

Any restrictions to use by non-academics: N/A.

## Data Availability

Source Code is available to download at https://github.com/nlm-malaria/MalariaScreener.

## Abbreviations

GUI: Graphical user interface
API: Application programming interface
RBC: Red blood cell
WBC: White blood cell
CNN: Convolutional neural network
PK: Primary key
